# Supplementary material for: Barriers and facilitators to Water, Sanitation and Hygiene (WaSH) practices in Southern Africa: A scoping review
Source: PLoS One. 2022 Aug 2;17(8):e0271726. doi: 10.1371/journal.pone.0271726 (PMC9345477; doi:10.1371/journal.pone.0271726)
Supplement: S1 Checklist — (PDF) [file pone.0271726.s001.pdf]

Preferred Reporting Items for Systematic reviews and Meta-Analyses extension for Scoping Reviews (PRISMA-ScR) Checklist

SECTION	ITEM	PRISMA-ScR CHECKLIST ITEM	REPORTED ON PAGE #
**TITLE**			
Title	1	Identify the report as a scoping review.	Barriers and facilitators to Water, Sanitation and Hygiene (WaSH) practices in Southern Africa: a scoping review.<br><br>Reported on page 1.
**ABSTRACT**			
Structured summary	2	Provide a structured summary that includes (as applicable): background, objectives, eligibility criteria, sources of evidence, charting methods, results, and conclusions that relate to the review questions and objectives.	**Background:** A healthy and a dignified life experience requires adequate water, sanitation, and hygiene (WaSH) coverage. However, inadequate WaSH resources remain a significant public health challenge in many communities in Southern Africa.<br><br>**Objectives:** The objective of this scoping review was to examine facilitators and barriers to effective WaSH practices in Southern Africa and to identified knowledge gaps on the same guided by the methodological framework for scoping.<br><br>**Eligibility criteria:** The review included articles describing interventions on WaSH practices in Southern Africa with a particular focus on facilitators and barriers. Articles included in the study were published in English language from 2010 to June 2021. Eighteen peer reviewed articles from Southern Africa satisfied the inclusion criteria for this review.<br><br>**Sources of evidence:** A systematic search of peer reviewed journal articles from 2010 – June 2021 was undertaken on Medline, PubMed, EbscoHost and Google Scholar from 2010 to June 2021 were searched using combinations of predefined search terms with Boolean operators.<br><br>**Charting methods:** In the data extraction phase, a total of 18 articles were selected based on the inclusion and exclusion criteria. PRISMA flow diagram showing steps followed to select articles was constructed. All records were downloaded using Zotero software and duplicates were removed. We created a data extraction table that captured the following information: authors, year of publication, objectives of

St. Michael's
Inspired Care.
Inspiring Science.

			the study, the type of the study, geographical location from where the studies were conducted and the summary of the main findings from each study.<br><br>**Results:** The general themes that emerged included geographical inequalities, climate change, investment on WaSH resources, low levels of knowledge on water borne diseases and ineffective local community engagement. Key facilitators to improved WaSH practices included improved WaSH infrastructure, effective local community engagement, increased latrine ownership by individual households and the development of social capital.<br><br>**Conclusion:** Water and sanitation are critical to ensuring healthy lifestyle. However, many people and communities in Southern Africa still lack access to safe water and improved sanitation facilities. Rural areas are the most affected by barriers to improved WaSH facilities compared to urban settings. Our review has shown that, the current WaSH conditions in Southern Africa do not equate to the improved WaSH standards described in the SDGs 6 on ensuring access to water and sanitation for all. Key barriers to improved WaSH practices identified include rurality, climate change, low investments to WaSH infrastructure, inadequate knowledge on water borne illnesses and lack of community engagement.<br><br>Reported on page 1
**INTRODUCTION**			
Rationale	3	Describe the rationale for the review in the context of what is already known. Explain why the review questions/objectives lend themselves to a scoping review approach.	Inadequate water, access to improved sanitation, and hygiene (WaSH) are global health challenges affecting about one-third of the world's population [8, 10]. Improved sanitation and hygiene are essential because they reduce environmental health risks [34]. Global diarrheal disease statistics show that more than one million annual deaths are related to poor WaSH practices as over one-third of the world's population do not have basic sanitation [1]. Although adequate WaSH coverage is critical for improving quality of life, globally about 2 billion people do not have access to clean water [2] and over 263 million people walk

			long distances to collect water from rivers, streams and lakes. At least 159 million people drink water from unsafe sources [2]. In Africa, about 70 percent of rural water schemes are non-functional or intermittently functional at any given time [3] resulting in compromised human wellbeing [4]. Due to poor WaSH practices in Africa, about 28 percent of the population in the region still practice open defecation [8]. Unsafe sanitation behaviours are responsible for around 775, 000 world deaths annually of which 5 percent are in low-income countries [8]. Universal, affordable, and sustainable access to WaSH is one of the key public health and development issues. Plans to improve WaSH coverage are instituted in Sustainable Development Goals (SDG) goal 6 which seeks to ensure availability and sustainable management of water and sanitation for all by 2030 [5]. Even though this SDG advocates for progressive reduction of inequalities related to hygiene and universal access to clean water and sanitation [5], continued inequalities in access to clean water and improved sanitation between rural and urban settings are still a challenge [6, 11, 12, 13].<br>Improved WaSH practices have the potential to reduce the prevalence of diseases such as schistosomiasis, cholera, diarrhea, polio, and typhoid which are prevalent in most sub-Saharan African countries. However, people still lack adequate information on WaSH leading to poor sanitation and hygiene practices. Southern Africa is among regions with very low rates of WaSH coverage in the world [5]. The provision of clean water to most rural communities in Southern Africa is insufficient and this exacerbates challenges associated with sanitation and hygiene [7]. For instance, hand washing is a cost effective and simple approach used for control of water-based infections and yet despite its simplicity and effectiveness it is not widely used [9].<br>Mitigating inequalities linked to access to WaSH is therefore critical. Understanding patterns of inequalities in WaSH practices, and how these are influenced by different facilitators and barriers is vital to providing effective interventions to mitigate inequalities in WaSH coverage in Southern Africa. Using a scoping review guided by the methodological framework for scoping, we

			examined facilitators and barriers to effective WaSH practices in Southern Africa and identified knowledge gaps on the same.<br><br>Reported on page 1
Objectives	4	Provide an explicit statement of the questions and objectives being addressed with reference to their key elements (e.g., population or participants, concepts, and context) or other relevant key elements used to conceptualize the review questions and/or objectives.	**Statement of the questions**<br>What are the facilitators and barriers to effective WaSH practices (concept) in Southern Africa (population and context)? What are the knowledge gaps that exist that are related to facilitators and barriers to effective WaSH practices in the region?<br><br>**Objectives**<br>a. To examine facilitators and barriers to effective WaSH practices in Southern Africa<br>b. To identify knowledge gaps on the same.<br><br>Reported on page 2
**METHODS**			
Protocol and registration	5	Indicate whether a review protocol exists; state if and where it can be accessed (e.g., a Web address); and if available, provide registration information, including the registration number.	**Protocol**<br>**Title**: Facilitators and barriers to water, sanitation, and hygiene (WaSH) practices in Southern Arica: a systematic review<br><br>**Purpose**: To examine facilitators and barriers to effective WaSH practices, and to identify research gaps on facilitators and barriers to effective WaSH practices in Southern Africa and describe direction for future research.<br><br>**Research question**: What are the facilitators and barriers to effective water, sanitation, and hygiene (WaSH) practices in Southern Africa?<br><br>**Specific objectives**<br>• To synthesize the existing information on WaSH practices in Southern Africa.<br>• To examine facilitators to effective WaSH practices in Southern Africa.<br>• To identify the existing research gaps on facilitators and barriers to effective WaSH practices in Southern African countries.<br><br>**Keywords**<br>• Facilitators and barriers<br>• Water, sanitation, and hygiene<br>• WaSH practices

- Southern Africa

Synonyms

- **Facilitators**: Drivers, motivators, enablers
- **Barriers**: Challenges, hindrances, obstacles

Data bases

- PubMed
- Medline
- EbscoHost
- Google Scholar

Search strings

We will conduct a systematic electronic search of peer reviewed journal articles from various databases including Google Scholar, PubMed, EbscoHost and Medline using the following keywords: “facilitators; barriers; water; sanitation; hygiene; WaSH practices and Southern Africa.” Using the keywords, we developed “index terms” from combining keywords and their synonyms and used the Boolean operators “AND”, “OR” and truncations to create search strings: “Water AND sanitation AND hygiene AND Facilitators (AND motivators) AND barriers (OR hindrances) AND WASH practices AND Southern Africa”.

Inclusion criteria

- Peer reviewed journal articles - qualitative, quantitative, and mixed methods studies on facilitators and barriers to WaSH practices in Southern Africa.
- Publications from 2010 – June 2021.
- Studies describing WaSH practices in Southern African countries (Angola, Botswana, Lesotho, Mozambique, Namibia, South Africa, Swaziland, Zambia, Zimbabwe).
- Publication language: English

Exclusion criteria

			• Publications before 2010.<br>• Studies describing WaSH practices in other continents outside Southern Africa.<br>• Publications in other language other than English.<br>• Reviews, e.g., systematic, scoping and meta-analysis.<br><br>**Not published, but uploaded as an additional document with submission.**
Eligibility criteria	6	Specify characteristics of the sources of evidence used as eligibility criteria (e.g., years considered, language, and publication status), and provide a rationale.	The review included:<br>a. Articles describing interventions on WaSH practices in Southern Africa with a particular focus on facilitators and barriers.<br><br>**Rationale**: The population we were interested in – communities in Southern Africa (population) who experience many water supply, sanitation and hygiene practices challenges (concept) because they are from the low-income countries (context) bracket.<br><br>b. Articles included in the study were published in English language.<br><br>**Rationale**: The choice of articles published in English was mainly for convenience (for all the authors who use English as their common second language).<br><br>c. Articles were published from 2010 to June 2021.<br><br>**Rationale**: The period between 2010 and June 2021 was decided because…<br><br>d. Articles were published in peer reviewed journals.<br><br>**Rationale**: The articles considered were those from peer reviewed journals because they are more credible due to rigorous review process they have gone through.<br><br>Reported on page 3
Information sources*	7	Describe all information sources in the search (e.g., databases with dates of coverage and contact with authors to identify additional sources), as well as the date	The following data bases were information sources in the search:<br>a. Medline<br>b. PubMed<br>c. EbscoHost<br>d. Google Scholar

		the most recent search was executed.	Dates of coverage: November 2020 – July 2021.<br><br>Contact with authors to identify additional sources: March 2021.<br><br>Most recent search was executed: July 2021<br><br>Reported on page 3
Search	8	Present the full electronic search strategy for at least 1 database, including any limits used, such that it could be repeated.	**PubMed:**<br>Search: **(((water) AND (sanitation)) AND (hygiene)) AND (southern africa) AND ((fha[Filter]) AND (journalarticle[Filter]) AND (fft[Filter]) AND (english[Filter]))** Filters: **Abstract, Full text, Journal Article, English, from 2010 - 2021**<br>(("water"[MeSH Terms] OR "water"[All Fields] OR "drinking water"[MeSH Terms] OR ("drinking"[All Fields] AND "water"[All Fields]) OR "drinking water"[All Fields] OR "watering"[All Fields] OR "waters"[All Fields] OR "water s"[All Fields] OR "watered"[All Fields] OR "waterer"[All Fields] OR "waterers"[All Fields] OR "waterings"[All Fields]) AND ("sanitation"[MeSH Terms] OR "sanitation"[All Fields] OR "sanitations"[All Fields] OR "sanitization"[All Fields] OR "sanitize"[All Fields] OR "sanitized"[All Fields] OR "sanitizer"[All Fields] OR "sanitizers"[All Fields] OR "sanitizing"[All Fields]) AND ("hygiene"[MeSH Terms] OR "hygiene"[All Fields] OR "hygienic"[All Fields] OR "hygienical"[All Fields] OR "hygienically"[All Fields] OR "hygienics"[All Fields] OR "hygienization"[All Fields]) AND ("africa, southern"[MeSH Terms] OR ("africa"[All Fields] AND "southern"[All Fields]) OR "southern africa"[All Fields] OR ("southern"[All Fields] AND "africa"[All Fields])) AND ("hasabstract"[All Fields] AND "journal article"[Publication Type] AND "loattrfull text"[Filter] AND "english"[Language])) AND ((fha[Filter]) AND (journalarticle[Filter]) AND (fft[Filter]) AND (english[Filter]) AND (2010:2021[pdat]))<br>**Translations**<br>**water:** "water"[MeSH Terms] OR "water"[All Fields] OR "drinking water"[MeSH Terms] OR ("drinking"[All Fields] AND "water"[All Fields]) OR "drinking water"[All Fields] OR "watering"[All Fields] OR "waters"[All Fields] OR "water's"[All Fields] OR "watered"[All Fields] OR "waterer"[All Fields] OR

St.Michael's
Inspired Care.
Inspiring Science.

			"waterers"[All Fields] OR "waterings"[All Fields]<br>**sanitation:** "sanitation"[MeSH Terms] OR "sanitation"[All Fields] OR "sanitations"[All Fields] OR "sanitization"[All Fields] OR "sanitize"[All Fields] OR "sanitized"[All Fields] OR "sanitizer"[All Fields] OR "sanitizers"[All Fields] OR "sanitizing"[All Fields]<br>**hygiene:** "hygiene"[MeSH Terms] OR "hygiene"[All Fields] OR "hygienic"[All Fields] OR "hygienical"[All Fields] OR "hygienically"[All Fields] OR "hygienics"[All Fields] OR "hygienization"[All Fields]<br>**southern africa:** "africa, southern"[MeSH Terms] OR ("africa"[All Fields] AND "southern"[All Fields]) OR "southern africa"[All Fields] OR ("southern"[All Fields] AND "africa"[All Fields])<br>**fha[Filter]:** hasabstract<br>**journalarticle[Filter]:** Journal Article[pt]<br>**fft[Filter]:** loattrfull text[subset]<br>**english[Filter]:** english [LA]<br><br>**Filters/ limits used**: Abstract, Full text, Journal Article, English, from 2010 - 2021
Selection of sources of evidence†	9	State the process for selecting sources of evidence (i.e., screening and eligibility) included in the scoping review.	Our electronic search from PubMed provided 1252 records, EbscoHost 62 records and 75 records from Google Scholar. The electronic title search provided a total of 1389 articles (Figure 1) from which 24 duplicates were removed. One thousand, three hundred and one (1301) articles were deemed illegible and were removed after screening their titles. Sixty-four (64) articles that remained were screened based on their relevance by abstracts and of these, twenty-one (21) articles were removed. Full-text screening for the remaining 43 articles was done and 30 articles were removed due to irrelevant focus and aims in relation to the objective of this review. Among those removed, one article covered a scope outside Southern Africa, another article used secondary data collected between 1995 - 2006 although the paper was published in 2015. One article was a working paper, and other excluded studies were reports, systematic and scoping reviews. We remained with 13 legible records deemed relevant. Five (5) additional records were identified from the reference lists of eligible articles and were included for full text review resulting in a total of 18 articles.

			Reported on page 3
Data charting process‡	10	Describe the methods of charting data from the included sources of evidence (e.g., calibrated forms or forms that have been tested by the team before their use, and whether data charting was done independently or in duplicate) and any processes for obtaining and confirming data from investigators.	We used inclusive approaches for data extraction. This is where all eligible data were included to avoid omitting findings of potential value to the synthesis. This approach is more comprehensive. Data extraction was done manually and independently. We then compared our independent charting and listed themes that we independently charted. Researchers used the charted data as a basis for creating a codebook. During the first stage, we familiarized ourselves with the individual charted data through reading data. This process allowed us to understand charted data and remarks made by individual investigators in the data. The generation of initial codes from the data was conducted during the second stage of data analysis process and we searched for themes emerging from data during the third stage. Themes created during the third stage were revised in the fourth staged, and were defined and named during the fifth stage. The final stage involved writing this review based on the data collected and focusing on the major themes that came out of the charted data.
Data items	11	List and define all variables for which data were sought and any assumptions and simplifications made.	• Financing<br>• Investment<br>• Population growth<br>• Knowledge (on healthy WASH practices)<br>• Community engagement<br>• Climate change<br>• WASH infrastructure<br>• Toilet ownership<br>• Social capital
Critical appraisal of individual sources of evidence§	12	If done, provide a rationale for conducting a critical appraisal of included sources of evidence; describe the methods used and how this information was used in any data synthesis (if appropriate).	Critical appraisal of included sources of evidence was conduct to appraise the quality of different study designs used in our included sources of evidence. We used Mixed Methods Appraisal Tool (MMAT) which includes quality criteria of five categories of study designs: (a) qualitative, (b) randomized controlled trial, (c) nonrandomized, (d) quantitative descriptive and (e) mixed methods studies. This appraisal tool focuses on core relevant methodological criteria and has five criteria per category of study design.<br><br>Reported on page 3

<table>
<tr><td>Synthesis of results</td><td>13</td><td>Describe the methods of handling and summarizing the data that were charted.</td><td>We used thematic analysis and inductive approach in handling and summarising data charted. Thematic analysis is a qualitative data analysis method that involves reading through data and identifying patterns and meaning in the data. The inductive approach is a systematic procedure for analyzing qualitative data in which the analysis is likely to be guided by specific evaluation objectives. Steps followed during thematic analysis were:
Step 1; Familiarization – Here we explored our data and got to know our data through reading the texts and taking notes. Step 2: Coding – We coded the data by highlighting sections, e.g. phrases and sentences in the text that related to our objectives. Step 3: Generating themes – We examined the codes we created in step 2 and identified patterns in the data, then started creating themes which were broader than codes. Step 4: Reviewing themes – We compared our themes to check what we could have missed and confirmed that our themes were really represented in the data. Step 5: Defining and naming themes – This is where we formulated what each theme meant and figured out how each of the themes helped us to understand the data. Step 6: Writing up the review.

Reported on page</td></tr>
<tr><td colspan="4">RESULTS</td></tr>
<tr><td>Selection of sources of evidence</td><td>14</td><td>Give numbers of sources of evidence screened, assessed for eligibility, and included in the review, with reasons for exclusions at each stage, ideally using a flow diagram.

Reported on page 4</td><td>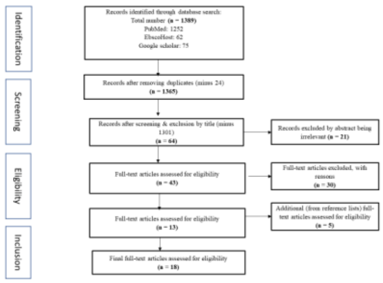
</td></tr>
<tr><td>Characteristics of sources of evidence</td><td>15</td><td>For each source of evidence, present characteristics for which data were charted and provide the citations.</td><td>We assessed all selected articles for quality using mixed methods appraisal tool (MMAT) [16]. MMAT is used as a tool to appraise the quality of different study designs [16]. For each study, we used scores ranging from 0 to 10, where 0 - 4 = “Low” quality, 5 – 7 = “Moderate” quality and 8 – 10 = “High” quality. The majority of the articles selected scored moderate. No studies scored “Low”, 17 articles scored “Moderate” and one article scored “High”. Indicators used for quality scores included: (a) a clear definition of the study objective and aim, (b) study</td></tr>
</table>

			design appropriate for stated aims, (c) justified sample size, (d) targeted population defined, (e) risk factor and outcome variables measured, (f) methods clearly described, (g) study results described, (h) discussions and conclusions justified, (i) study limitations discussed and (j) ethical approval for the study attained.<br><br>Reported on page 3
Critical appraisal within sources of evidence	16	If done, present data on critical appraisal of included sources of evidence (see item 12).	For each study, we used scores ranging from 0 to 10, where 0 - 4 = "Low" quality, 5 – 7 = "Moderate" quality and 8 – 10 = "High" quality. The majority of the articles selected scored moderate.<br><br>**Summary data:**<br>• 0 studies scored "Low"<br>• 17 articles scored "Moderate"<br>• 1 article scored "High".<br><br>**NB** – A detailed table is attached as one of additional document - Appraisal for the quality of studies.<br><br>Reported on page 3
Results of individual sources of evidence	17	For each included source of evidence, present the relevant data that were charted that relate to the review questions and objectives.	**Results of individual sources of evidence:**<br><br>1. **Source**: Tubatsi, G., Bonyongo, M.C. & Gondwe, M. (2015)<br><br>**Title**: Water use practices, water quality, and households' diarrheal encounters in communities along the Boro-Thamalakane-Boteti river system, Northern Botswana<br><br>**Relevant data charted**<br><br>**Facilitators to improved WaSH practices**<br><br>• Water quality: Clean water promotes healthy WaSH behaviours.<br>• Water storage at home – Improved ways to store water at home facilitate improved WaSH practices in a home setting.<br>• Integrated control programs focusing on improving quality of water both at source and point of use.<br>• Promotion of improved hygiene practices.

			**Barriers to improved WaSH practices**<br>• Water quality: Contaminated water hinders healthy WaSH practices.<br>• Water storage at home – Poor water storage facilities at home compromise sanitation and hygiene practices at home.<br>• Inadequate awareness created on behaviours that promote improved hygiene practices.<br><br>**2. Source**: McGill, B.M., Altchenko, Y., Hamilton, S.K., Kenabatho, P.K., Sylvester, S.R. & Villholth, K.G. (2019).<br><br>**Title**: Complex interactions between climate change, sanitation, and groundwater quality: a case study from Ramotswa, Botswana.<br><br>**Relevant data charted:**<br><br>**Facilitators to improved WaSH practices**<br>• Economic activity: Economic activity depends mainly on political willingness by the government.<br><br>**Barriers to improved WaSH practices**<br>• Demographics – High population density compromises improved WaSH practices in Southern African countries characterized by poor and lack of WaSH infrastructure to service high population.<br>• Economic activity – Declining/weak economies in most Southern African countries hinders improvements in WaSH facilities thereby hindering developments and improvements for WaSH practices.<br>• Climate change – Has brought many challenges, e.g. drought which negatively affects reliability of water supply in the region, resulting in poor and compromised WaSH practices.<br>• Land use – Human activities on land, e.g. pollution has led to

			climate experiences which have brought or resulted in droughts.<br><br>3. **Source**: Mlenga, D.H. (2016).<br><br>**Title**: Towards Community Resilience, Focus on a Rural Water Supply, Sanitation and Hygiene Project in Swaziland.<br><br>**Relevant data charted:**<br><br>**Facilitators to improved WaSH practices**<br><br>• The WaSH interventions implemented by the NGOs.<br>• Improved access and availability of potable water.<br>• Improved knowledge, changed attitudes and practices towards hygiene and sanitation.<br><br>**Barriers to improved WaSH practices**<br><br>• Drought.<br>• Local community's resilience to the prevailing WaSH challenges.<br>• Inadequate/lack of WaSH infrastructure.<br>• Low investment in WaSH infrastructure.<br><br>4. **Source**: Gwimbi, P. (2011).<br><br>**Title**: The microbial quality of drinking water in Manonyane community: Maseru District (Lesotho).<br><br>**Relevant data charted:**<br><br>**Facilitators to improved WaSH practices**<br><br>• Prompt intervention to mitigate the potential health impact of water-borne diseases in the community.<br>• A proper sanitary survey and implementation of water and sanitation projects in the community.<br><br>**Barriers to improved WaSH practices**<br><br>• Pollution on the physical environment – Land pollution.<br>• Poor source water protection.

			• Poor sanitation and low level of hygiene practices.<br>• Lack of monitoring and healthcare awareness.<br><br>5. **Source**: Gwimbi, P., George, M. & Ramphalile, M. (2019).<br><br>**Title**: Bacterial contamination of drinking water sources in rural villages of Mohale Basin, Lesotho: exposures through neighbourhood sanitation and hygiene practices.<br><br>**Relevant data charted:**<br><br>**Facilitators to improved WaSH practices**<br><br>• Source water protection status.<br>• Community-led sanitation and hygiene education.<br>• Improved water source protection.<br><br>**Barriers to improved WaSH practices**<br><br>• Poor and lack of source water protection.<br>• Contamination of water sources by e.coli.<br>• Contamination of water sources with faeces.<br>• Poor neighbourhood sanitation and hygiene condition.<br><br>6. **Source**: Chunga, R.M., Ensink, J.H.J., Jenkins, M.W. & Brown, J. (2016).<br><br>**Title**: Adopt or Adapt: Sanitation Technology Choices in Urbanizing Malawi.<br><br>**Relevant data charted:**<br><br>**Facilitators to improved WaSH practices**<br><br>• Effective pit emptying services.<br>• Construction of new pit latrines with slabs.<br>• Adaptation of locally promoted, novel sanitation technology known as ecological sanitation (ecosan).<br><br>**Barriers to improved WaSH practices**

			• Lack of pit emptying services.<br>• Concerns about space for replacing pit latrines.<br>• Reluctance to unknown technology in pit latrine construction.<br><br>**7.** Shiras, T., Cumming, O., Brown, J., Muneme, B., Nala, R. & Dreibelbis, R. (2018).<br><br>**Title**: Shared Sanitation Management and the Role of Social Capital: Findings from an Urban Sanitation Intervention in Maputo, Mozambique.<br><br>**Relevant data charted:**<br><br>**Facilitators to improved WaSH practices**<br><br>• Developing social capital within small community units.<br>• WASH interventions employing effective collective action strategies to disseminate lessons and share behavior change tactics, e.g. electing a compound leader to implement and oversee adherence to latrine management strategies.<br>• Increased latrine ownership by individual households.<br>• Collective decision-making for shared larine users.<br>• Creating monthly financial contribution to help with ongoing latrine maintenance costs or cleaning supplies.<br>• Simple, low cost interventions informed by modern behavioral science to provide replicable approaches for increasing social capital or finding mechanisms for latrine management that rely less on complex social processes.<br><br>**Barriers to improved WaSH practices**<br><br>• Shared sanitation – It is difficult to maintain hygiene in shared sanitation facilities.<br>• Shared toilet facilities.

			• Poverty and lack of funds to contribute for consistency in proper maintenance of shared latrines.<br><br>**8. Source**: Hans-Joachim, M., Mosch, S. & Harter, M. (2018).<br><br>**Title**: Is Community-Led Total Sanitation connected to the rebuilding of latrines? Quantitative evidence from Mozambique.<br><br>**Relevant data charted:**<br><br>**Facilitators to improved WaSH practices**<br><br>• Community-Led Total Sanitation (CLTS).<br>• Latrine rebuilding depends on education, soil conditions, social cohesion, and a feeling of being safe from diarrhoea, the perception that many other community members own a latrine, and high confidence in personal ability to repair or rebuild a broken latrine.<br>• Social and psychosocial factors.<br><br>**Barriers to improved WaSH practices**<br><br>• Heavy rains hit the north of Mozambique and many latrines collapsed.<br>• Minimum/lack of cooperation by some community members in CLTS.<br><br>**9. Source**: Lewis, E.W., Nguza, S. & Selma, L. (2018).<br><br>**Title**: Assessment of accessibility of safe drinking water: A case study of the Goreangab informal settlement, Windhoek, Namibia.<br><br>**Relevant data charted:**<br><br>**Facilitators to improved WaSH practices**<br><br>• Incorporation of an integrated water resource management framework and a public–private partnership to improve the settlement's water supply management.

			**Barriers to improved WaSH practices**<br><br>• Informal settlements.<br>• Poor water accessibility.<br>• Long distances to water sources.<br>• Water affordability.<br>• People's high reliance on contaminated water for cooking and drinking.<br>• The inability of the municipality to meet the demands of migrants flocking in search for better opportunities.<br><br>**10. Source**: Abia, A.L.K., Schaefer, L., Ubomba-Jaswa, E., & Le Roux, W. (2017).<br><br>**Title**: Abundance of Pathogenic Escherichia coli Virulence-Associated Genes in Well and Borehole Water Used for Domestic Purposes in a Peri-Urban Community of South Africa.<br><br>**Relevant data charted:**<br><br>**Facilitators to improved WaSH practices**<br><br>• Community engagement/involvement.<br>• Protected water sources.<br>• Water infrastructure<br><br>**Barriers to improved WaSH practices**<br><br>• Absence of water infrastructure<br>• Alternative water sources, e.g. unprotected wells, dams and rivers.<br>• Poor maintenance of sanitation facilities.<br>• Pollution<br>• Pathogenic E. coli strains.<br>• Poor community engagement.<br><br>**11.** Source: Sibiya, J.E. & Gumbo, J.R. (2013).<br><br>**Title**: Knowledge, Attitude and Practices (KAP) Survey on Water, Sanitation and Hygiene in Selected Schools in Vhembe District, Limpopo, South Africa.<br><br>**Relevant data charted:**

			**Facilitators to improved WaSH practices**<br><br>• The high level of knowledge about waterborne diseases.<br>• Positive attitude and improved practices on hygiene.<br>• Urban settings.<br>• Proper handwashing facilities.<br>• Clear borehole water quality though the microbial quality was unknown.<br>• Adequate water sources.<br><br>**Barriers to improved WaSH practices**<br><br>• Inadequate knowledge on transmission routes of waterborne diseases.<br>• Lack of knowledge in relation to water-based diseases and their prevention.<br>• Lack of soap at handwashing facilities.<br>• Inadequate water supply and sanitation facilities, e.g. in rural settings/schools.<br>• No handwashing areas and no sanitary bins for girls.<br>• Some schools had toilets with broken toilet doors offering no privacy.<br>• Inadequate water sources.<br><br>**12. Source**: Nefale, A.D., Kamika, I., Obi, C.I. & Momba, M.N.B. (2017).<br><br>**Title**: The Limpopo Non-Metropolitan Drinking Water Supplier Response to a Diagnostic Tool for Technical Compliance.<br><br>**Relevant data charted:**<br><br>Facilitators to improved WaSH practices<br><br>**Barriers to improved WaSH practices**<br><br>• Compliance of small water treatment plants with accepted drinking water quality standards and management norms is still a challenge in the rural areas of South Africa.<br>• Poor condition of laboratory equipment and operations.

			• Shortage of staff, especially skilled personnel.<br>• Lack of measuring instruments/laboratory equipment, chemicals.<br>• Insufficient funds.<br><br>**13.** Source: Tidwell, J.B., Chipungu, J., Chilengi, R., Curtis, V. & Aunger, R. (2019).<br><br>**Title**: Theory-driven formative research on on-site, shared sanitation quality improvement among landlords and tenants in peri-urban Lusaka, Zambia.<br><br>**Relevant data charted:**<br><br>**Facilitators to improved WaSH practices**<br><br>• Shared, on-site sanitation maintenance and improvement behaviors.<br>• Consumer-driven, sustainable improvements investments in toilet improvements.<br>• Introducing better shared cleaning systems.<br><br>**Barriers to improved WaSH practices**<br><br>• Poor coordination among tenants – shared sanitation facilities.<br>• Lack of communication between users of shared sanitation facilities, e.g. landlords and tenants.<br><br>**14. Source**: Psutka, R., Peletz, R., Michelo, S., Kelly, P. & Clasen, T. (2020).<br><br>**Title**: Assessing the Microbiological Performance and Potential Cost of Boiling Drinking Water in Urban Zambia.<br><br>**Relevant data charted:**<br><br>**Facilitators to improved WaSH practices**<br><br>• Safe-storage practices to minimize recontamination.<br><br>**Barriers to improved WaSH practices**

			• Over-reporting and inconsistent compliance to 'cleaning' water for drinking.<br>• Lack of residual protection and unsafe storage and handling.<br>• Cost of boiling - The potential cost of fuel or electricity for boiling.<br><br>**15. Source**: Thys, S., Mwape, K.E., Lefèvre, P., Dorny, P., Marcotty, T., Phiri, A.M., Phiri, I.K. & Gabriël, S. (2015).<br><br>**Title**: Why Latrines Are Not Used: Communities' Perceptions and Practices Regarding Latrines in a Taenia solium Endemic Rural Area in Eastern Zambia.<br><br>**Relevant data charted:**<br><br>**Facilitators to improved WaSH practices**<br><br>• A "people-centered" preventive approach that addresses both the perception of the disease and its management.<br>• Control strategies directed to the patterns of people's behavior associated with the phases of transmission of the disease.<br>• People's perceptions, knowledge and reported behaviors regarding the use and the construction of latrines.<br>• Seeking privacy and taboos were both identified as the key factors influencing the possession and use of sanitation facilities.<br>• Latrine promotion messages that are not only focused on health benefits.<br>• Anthropological studies for an in-depth understanding of sanitation practices within particular contexts in order to enhance the design of adapted interventions.<br><br>**Barriers to improved WaSH practices**<br><br>• The existing challenges of cysticercosis control in endemic regions.

			• People's perceptions, knowledge and reported behaviors regarding the use and the construction of latrines.<br><br>**16. Source**: Tidwell, J.B., Chipungu, J., Bosomprah, S., Aunger, R., Curtis, V. & Chilengi, R. (2019).<br><br>**Title**: Effect of a behaviour change intervention on the quality of peri-urban sanitation in Lusaka, Zambia: a randomised controlled trial.<br><br>**Relevant data charted:**<br><br>**Facilitators to improved WaSH practices**<br><br>• Improved quality of toilets provided.<br>• Willingness to pay for quality improvements of toilets.<br>• Improved WaSH practices.<br><br>**Barriers to improved WaSH practices**<br><br>• Toilets shared by multiple households.<br><br>• The poor quality of toilet provision.<br><br>**17. Source**: Yeboah-Antwi, K., MacLeod, W.B., Biemba, G., Sijenyi, P., Hohne, A., Verstraete, L., McCallum, C.M. & Hamer, D.H. (2019).<br><br>**Title**: Improving Sanitation and Hygiene through Community-Led Total Sanitation: The Zambian Experience.<br><br>**Relevant data charted:**<br><br>**Facilitators to improved WaSH practices**<br><br>• Community-led total sanitation implementation.<br>• Access to improved sanitation facilities.<br>• Reduced open defecation.<br>• Improved handwashing practices.<br>• Enhanced investment in sanitation and hygiene promotion.

			**Barriers to improved WaSH practices**<br><br>• Lack of access to toilet and hygiene facilities.<br>• Inadequate investment on sanitation and hygiene promotion programs.<br>• Reluctance in behaviour change, e.g. open defecation practices.<br><br>**18. Source**: Ncube, F., Kanda, A., Chahwanda, M., Margaret Macherera, M. & Ngwenya, B. (2020).<br><br>**Title**: Predictors of hand hygiene behaviours among primary and secondary school children in a rural district setting in Zimbabwe: a cross-sectional epidemiologic study.<br><br>**Relevant data charted:**<br><br>**Facilitators to improved WaSH practice**<br><br>• Investment in hand hygiene behaviour change processes.<br>• WaSH promotion campaigns among school children.<br>• Empowerment of WaSH clubs in schools.<br><br>**Barriers to improved WaSH practices**<br><br>• Lack of investment on WaSH facilities<br>• Lack of awareness on healthy WaSH practices.<br>• Inadequate investment in WaSH infrastructure and facilities.<br><br>Reported from page 3
Synthesis of results	18	Summarize and/or present the charting results as they relate to the review questions and objectives.	Our electronic search from PubMed provided 1252 records, EbscoHost 62 records and 75 records from Google Scholar. The electronic title search provided a total of 1389 articles (Figure 1) from which 24 duplicates were removed. One thousand, three hundred and one (1301) articles were deemed illegible and were removed after screening their titles. Sixty-four (64) articles that remained were screened based on their relevance by abstracts and of these, twenty-one (21) articles were removed. Full-text screening for the remaining 43 articles was done and 30 articles were removed due to

			irrelevant focus and aims in relation to the objective of this review. Among those removed, one article covered a scope outside Southern Africa, another article used secondary data collected between 1995 - 2006 although the paper was published in 2015. One article was a working paper, and other excluded studies were reports, systematic and scoping reviews. We remained with 13 legible records deemed relevant. Five (5) additional records were identified from the reference lists of eligible articles and were included for full text review resulting in a total of 18 articles (PRISMA flow diagram).<br><br>Reported on page 3
**DISCUSSION**			
Summary of evidence	19	Summarize the main results (including an overview of concepts, themes, and types of evidence available), link to the review questions and objectives, and consider the relevance to key groups.	Our review of published articles on WaSH practices in Southern Africa identified and analysed facilitators and barriers to effective implementation of WaSH. The major themes that emerged as key facilitators to WaSH practices in this review were: (1) increased investment on WaSH infrastructure, (2) effective local community engagement, (3) increased latrine/toilet ownership by individual households, and (4) development of social capital within small community units. The following barrier themes emerged from the analysis: (1) geographical inequalities, (2) climate change, (3) low investment on WaSH infrastructure, (4) low knowledge levels on waterborne diseases, (5) ineffective local community engagement.<br><br>Reported on page 20
Limitations	20	Discuss the limitations of the scoping review process.	We reviewed articles from almost all the countries in Southern Africa but limited the search of articles to only those published in English thus possibly missing experiences from some countries in the region. We may also have missed some critical literature because we only focused on literature published in peer reviewed journals. We acknowledge that the application of filters during database search may have excluded other studies that could have been relevant in the review. Despite these limitations, we believe that our search strategy was comprehensive, and that we reviewed relevant literature in public health and the subject matter we explored.<br><br>Reported on page 22

Conclusions	21	Provide a general interpretation of the results with respect to the review questions and objectives, as well as potential implications and/or next steps.	Water and sanitation are critical to ensuring healthy lifestyle. However, many people and communities in Southern Africa still lack access to safe water and improved sanitation facilities. Rural areas are the most affected by barriers to improved WaSH facilities compared to urban settings. Studies focusing on the mitigation of the existing inequalities related to WaSH developments should be conducted. Our review has shown that, the current WaSH conditions in Southern Africa do not equate to the improved WaSH standards described in the SDGs 6 on ensuring access to water and sanitation for all. Key barriers to improved WaSH practices identified include rurality, climate change, low investments to WaSH infrastructure, inadequate knowledge on water borne illnesses and lack of community engagement. The review also identified facilitators to WaSH practices comprising social capital development, increased latrine ownership, effective local community engagement and increased investment to WaSH infrastructure. A knowledge gap exists in continued monitoring of progress in facilitators and barriers to improved WaSH practices in the region. There is also a gap in literature on solutions to mitigating existing barriers to improved WaSH practices in Southern Africa.<br><br>Reported on page 22
**FUNDING**			
Funding	22	Describe sources of funding for the included sources of evidence, as well as sources of funding for the scoping review. Describe the role of the funders of the scoping review.	This research was funded by the National Institute for Health Research (NIHR) Global Health Research programme (16/136/33), UK and the University of KwaZulu-Natal. We also acknowledge University Administration Support Program (UASP) funding for this manuscript.<br><br>Reported on page 22
References	23	List of references	1. Ginja, S., Gallagher, S. & Keenan, M. (2019) Water, sanitation and hygiene (WASH) behaviour change research: why an analysis of contingencies of reinforcement is needed, *International Journal of Environmental Health Research*, DOI: 10.1080/09603123.2019.1682127.

			2. World Health Organization. (2017). Progress on drinking water, sanitation and hygiene. [accessed 2020 Nov 22]. http://www.wipo.int/amc/en/mediation/rules<br>3. Valcourt, N., Javernick-Will, A., Walters, J. & Linden, K. (2020). System Approaches to Water, Sanitation, and Hygiene: A Systematic Literature Review. *International Journal of Environmental Research and Public Health*, 17, 702.<br>4. World Health Organization. (2014). *Public Health and Environment in the African Region: Report on the Work of WHO, 2012–2013*. World Health Organization Regional Office for Africa, Brazzaville.<br>5. Roche, R., Bain, R. & Cumming, O. (2017). A long way to go - Estimates of combined water, sanitation, and hygiene coverage for 25 sub-Saharan African countries. *PLoS ONE*, 12(2): e0171783.<br>6. Mackinnon, E., Ayah, R., Taylor, R., Owor, M., Ssempebwa, J., Olago, D., Kubalako, R., Dia, A.T., Gaye, C., Campos, L.C. & Fottrell, E. (2019). 21st century research in urban WASH and health in sub-Saharan Africa: methods and outcomes in transition, *International Journal of Environmental Health Research*, 29:4, 457-478.<br>7. Kamara, J.K., Galukande, M., Maeda, F., Luboga, S. and Renzaho, AM.N. (2017). Understanding the Challenges of Improving Sanitation and Hygiene Outcomes in a Community Based Intervention: A Cross-Sectional Study in Rural Tanzania. *International Journal of Environmental Research and Public Health*, 14, 60.<br>8. Ritchie, H. & Roser, M. (2019). Our World in Data.<br>9. Bulleda, N., Poppeb, K., Ramatsistic, K., Sitsulac, L., Winegard, G., Gumboc, J.,

			Dillinghame, R. and Smith, J. (2017). Assessing the environmental context of hand washing among school children in Limpopo, South Africa. *Water Int*, 42(5): 568–584.<br>10. Omidakhsh, N. & von Ehrenstein, O.S. (2021). Improved Water, Sanitation and Utilization of Maternal and Child Health Services in South Asia—An Analysis of Demographic Health Surveys. *International Journal of Environmental Research and Public Health*, 18(14):7667.<br>11. Roche, R., Bain, R. & Cumming, O. (2017). A long way to go – Estimates of combined water, sanitation and hygiene coverage for 25 sub-Saharan African countries. *PLoS ONE*, 12(2): e0171783.<br>12. Bouzid, M., Cumming, O. & Hunter, P.R. (2018). What is the impact of water sanitation and hygiene in healthcare facilities on care seeking behaviour and patient satisfaction? A systematic review of the evidence from low-income and middle-income countries. *BMJ Global Health*, 3: e000648.<br>13. McMichael, C. (2019). Water, Sanitation and Hygiene (WASH) in Schools in Low-Income Countries: A Review of Evidence of Impact. *International Journal of Environmental Research and Public Health*, 16(3):359.<br>14. Munn, Z., Peters, M.D.J, Stern, C., Tufanaru, C., McArthur, A. & Aromataris, E. (2018). Systematic review or scoping review? Guidance for authors when choosing between a systematic or scoping review approach. *BMC Med Res Methodol*, 18, 143.<br>15. Arksey, H, O'Malley, L. (2005). Scoping studies: towards a methodological framework. *Int J Soc Res Methodol*, 8(1):19–32.<br>16. Hong, Q.N., Fabregues, S., Bartlett, G., Boardman, F., Cargod, M., Dagenais, P., Gagnon, M., Griffiths,

			F., Nicolau, B., O'cathain, A., Rousseau, M., Vedel, I., Pluye, P. (2018). The Mixed Methods Appraisal Tool (MMAT) version 2018 for information professionals and researchers. *Education for Information*, 34 (4), 285-291.<br>17. Tubatsi, G., Bonyongo, M.C. & Gondwe, M. (2015). Water use practices, water quality, and households' diarrheal encounters in communities along the Boro-Thamalakane-Boteti river system, Northern Botswana. *Journal of Health, Population and Nutrition*, 33:21.<br>18. Gwimbi, P., George, M. & Ramphalile, M. (2019). Bacterial contamination of drinking water sources in rural villages of Mohale Basin, Lesotho: exposures through neighbourhood sanitation and hygiene practices. *Environ Health Prev Med*, 24, 33.<br>19. Hans-Joachim, M., Mosch, S. & Harter, M. (2018). Is Community-Led Total Sanitation connected to the rebuilding of latrines? Quantitative evidence from Mozambique. *PLoS ONE*, 13(5): e0197483.<br>20. Abia, A.L.K., Schaefer, L., Ubomba-Jaswa, E., Le Roux, W. (2017). Abundance of Pathogenic Escherichia coli Virulence-Associated Genes in Well and Borehole Water Used for Domestic Purposes in a Peri-Urban Community of South Africa. *International Journal of Environmental Research and Public Health*, 14(3):320.<br>21. Nefale, A.D., Kamika, I., Obi, C.L, Momba, M.N. (2017). The Limpopo Non-Metropolitan Drinking Water Supplier Response to a Diagnostic Tool for Technical Compliance. *International Journal of Environmental Research and Public Health*, 14(7):810.<br>22. Psutka, R., Peletz, R., Michelo, S., Kelly, P. & Clasen, T. (2011).

			Assessing the Microbiological Performance and Potential Cost of Boiling Drinking Water in Urban Zambia. *Environmental Science & Technology*, 45 (14), 6095-6101.<br>23. Shiras, T., Cumming, O., Brown, J., Muneme, B., Nala, R. & Dreibelbis, R. (2018).<br>24. Tidwell, J.B., Chipungu, J., Bosomprah, S., Aunger, R., Curtis, V. & Chilengi, R. (2019). Effect of a behaviour change intervention on the quality of peri-urban sanitation in Lusaka, Zambia: a randomised controlled trial. *The Lancet Planetary Health*, 3(4), e187-e196.<br>25. Thys, S., Mwape, K.E., Lefèvre, P., Dorny, P., Marcotty, T., Phiri, A.M., Phiri, I.K. & Gabriël, S. (2015). Why Latrines Are Not Used: Communities' Perceptions and Practices Regarding Latrines in a Taenia solium Endemic Rural Area in Eastern Zambia. *PLoS Negl Trop Dis*, 9(3): e0003570.<br>26. Ncube, F., Kanda, A., Chahwanda, M., Margaret Macherera, M. & Ngwenya, B. (2020). Predictors of hand hygiene behaviours among primary and secondary school children in a rural district setting in Zimbabwe: a cross-sectional epidemiologic study. *Journal of Water, Sanitation and Hygiene for Development*, 10 (4): 851–861.<br>27. McGill, B.M., Altchenko, Y., Hamilton, S.K., Kenabatho, P.K., Sylvester, S.R. & Villholth, K.G. (2019). Complex interactions between climate change, sanitation, and groundwater quality: a case study from Ramotswa, Botswana. *Hydrogeology Journal volume*, 27, 997–1015.<br>28. Mlenga, D.H. (2016). "Towards Community Resilience, Focus on a Rural Water Supply, Sanitation and Hygiene Project in Swaziland." *American Journal of Rural Development*, 4(4): 85-92.<br>29. Gwimbi, P. (2011). The microbial quality of drinking water in

			Manonyane community: Maseru District (Lesotho). *Afr Health Sci*,11(3):474-80.<br>30. Chunga, R.M., Ensink, J.H.J., Jenkins, M.W. & Brown, J. (2016) Adopt or Adapt: Sanitation Technology Choices in Urbanizing Malawi. *PLoS ONE*, 11(8): e0161262.<br>31. Lewis, E.W., Siyambango, N. & Lendelvo, S. (2018). Assessment of accessibility of safe drinking water: A case study of the Goreangab informal settlement, Windhoek, Namibia. *Water Practice and Technology*, 13 (4): 871–878.<br>32. Sibiya, J.E. & Gumbo, J.R. (2013). Knowledge, attitude and practices (KAP) survey on water, sanitation and hygiene in selected schools in Vhembe District, Limpopo, South Africa. *Int J Environ Res Public Health*, 4;10(6):2282-2295.<br>33. Yeboah-Antwi, K., MacLeod, W.B., Biemba, G., Sijenyi, P., Höhne, A., Verstraete, L., McCallum, C.M. & Hamer, D.H. (2019). Improving Sanitation and Hygiene through Community-Led Total Sanitation: The Zambian Experience. *Am J Trop Med Hyg*,100(4):1005-1012.<br>34. Angoua, E.L.E., Dongo, K., Templeton, M.R., Zinsstag, J. & Bonfoh, B. (2018). Barriers to access improved water and sanitation in poor peri-urban settlements of Abidjan, Côte d'Ivoire. *PLoS ONE*, 13(8): e0202928.<br>35. McMichael, C. (2018). Toilet Talk: Eliminating Open Defecation and Improved Sanitation in Nepal. *Medical Anthropology*, 37:4, 294-310.<br>36. Ajemu, K.F., Desta, A.A., Berhe, A.A., Woldegebriel, A.G. & Bezabih, N.M. (2020). Latrine Ownership and Its Determinants in Rural Villages of Tigray, Northern Ethiopia: Community-Based Cross-Sectional Study. *Journal of Environmental and Public Health*, 2123652, 8.

			37. Howard, G., Calow, R., Macdonald, A. & Bartram, J. (2016). Climate Change and Water and Sanitation: Likely Impacts and Emerging Trends for Action. *Annual Review of Environment and Resources*, 41:1, 253-276.<br>38. Mulopo, C., Kalinda, C. & Chimbari, M.J. (2020). Contextual and Psychosocial Factors Influencing the Use of Safe Water Sources: A Case of Madeya Village, uMkhanyakude District, South Africa. *Int. J. Environ. Res. Public Health*, 17, 1349.<br>39. O'Mara-Eves, A., Brunton, G., Oliver, S., Kavanagh, J., Jamal, F. & Thomas, J. (2015). The effectiveness of community engagement in public health interventions for disadvantaged groups: a meta-analysis. *BMC Public Health*, 15, 129.<br>40. Yeboah-Antwi, K., MacLeod, W.B., Biemba, G., Sijenyi, P., Höhne, A., Verstraete, L., McCallum, C.M. & Hamer, D.H. (2019). Improving Sanitation and Hygiene through Community-Led Total Sanitation: The Zambian Experience. *Am J Trop Med Hyg*. 100(4):1005-1012.<br>41. Pullan, R.L., Freeman, M.C., Gething, P.W. & Brooker, S.J. (2014). Geographical Inequalities in Use of Improved Drinking Water Supply and Sanitation across Sub-Saharan Africa: Mapping and Spatial Analysis of Cross-sectional Survey Data. *PLoS Med*, 11(4): e1001626.

JBI = Joanna Briggs Institute; PRISMA-ScR = Preferred Reporting Items for Systematic reviews and Meta-Analyses extension for Scoping Reviews.
* Where *sources of evidence* (see second footnote) are compiled from, such as bibliographic databases, social media platforms, and Web sites.
† A more inclusive/heterogeneous term used to account for the different types of evidence or data sources (e.g., quantitative and/or qualitative research, expert opinion, and policy documents) that may be eligible in a scoping review as opposed to only studies. This is not to be confused with *information sources* (see first footnote).
‡ The frameworks by Arksey and O'Malley (6) and Levac and colleagues (7) and the JBI guidance (4, 5) refer to the process of data extraction in a scoping review as data charting.

§ The process of systematically examining research evidence to assess its validity, results, and relevance before using it to inform a decision. This term is used for items 12 and 19 instead of "risk of bias" (which is more applicable to systematic reviews of interventions) to include and acknowledge the various sources of evidence that may be used in a scoping review (e.g., quantitative and/or qualitative research, expert opinion, and policy document).

From: Tricco AC, Lillie E, Zarin W, O'Brien KK, Colquhoun H, Levac D, et al. PRISMA Extension for Scoping Reviews (PRISMAScR): Checklist and Explanation. Ann Intern Med. 2018;169:467–473. doi: 10.7326/M18-0850.
