# Supplementary material for: Barriers and facilitators to Water, Sanitation and Hygiene (WaSH) practices in Southern Africa: A scoping review
Source: PLoS One. 2022 Aug 2;17(8):e0271726. doi: 10.1371/journal.pone.0271726 (PMC9345477; doi:10.1371/journal.pone.0271726)
Supplement: S1 File — (DOCX) [file pone.0271726.s002.docx]

**Search strategy - PubMed**

**Topic**: Barriers and facilitators to Water, Sanitation and Hygiene (WaSH) practices in Southern Africa: a scoping review

**Keywords**

|  | **Synonyms** |
| --- | --- |
| - Barriers - Facilitators - WASH practices - Southern Africa | Hindrances  Motivators |

**Using the Boolean operators “AND”, “OR” and truncations to create search strings:**

- “*Water AND sanitation AND hygiene AND Facilitators (AND motivators) AND barriers (OR hindrances) AND WASH practices AND Southern Africa”*

|  | **Number of hits** |
| --- | --- |
| **Search**: ((water*[tw]) AND (sanitation*[tw])) AND (hygiene*[tw])  **Filters**: Abstract, Free full text, Full text, Meta-Analysis, Review, Systematic Review, in the last 10 years, English  ("water*"[Text Word] AND "sanitation*"[Text Word] AND "hygiene*"[Text Word]) AND ((y_10[Filter]) AND (ffrft[Filter]) AND (fha[Filter]) AND (meta-analysis[Filter] OR review[Filter] OR systematicreview[Filter]) AND (fft[Filter]) AND (english[Filter])) | 193 |
| **Search**: (((((water*[tw]) OR (drinking water supplier*[tw])) AND (sanitation*[tw])) OR (shared sanitation*[tw])) AND (hygiene*[tw])) OR (cleanliness*)  **Filters**: Abstract, Free full text, Full text, Meta-Analysis, Review, Systematic Review, in the last 10 years, English  ((((("water*"[Text Word] OR "drinking water supplier*"[Text Word]) AND "sanitation*"[Text Word]) OR "shared sanitation*"[Text Word]) AND "hygiene*"[Text Word]) OR "cleanliness*"[All Fields]) AND ((y_10[Filter]) AND (ffrft[Filter]) AND (fha[Filter]) AND (meta-analysis[Filter] OR review[Filter] OR systematicreview[Filter]) AND (fft[Filter]) AND (english[Filter])) | 286 |
| **Search**: (barriers*) AND (WASH practices*[tw])  **Filters**: Abstract, Free full text, Full text, Meta-Analysis, Review, Systematic Review, in the last 10 years, English  ("barriers*"[All Fields] AND "wash practices*"[Text Word]) AND ((y_10[Filter]) AND (ffrft[Filter]) AND (fha[Filter]) AND (meta-analysis[Filter] OR review[Filter] OR systematicreview[Filter]) AND (fft[Filter]) AND (english[Filter])) | 1 |
| **Search**: ((facilitators*[tw]) OR (sanitation*[tw])) OR (shared sanitation*[tw])  **Filters**: Abstract, Free full text, Full text, Meta-Analysis, Review, Systematic Review, in the last 10 years, English  ("facilitators*"[Text Word] OR "sanitation*"[Text Word] OR "shared sanitation*"[Text Word]) AND ((y_10[Filter]) AND (ffrft[Filter]) AND (fha[Filter]) AND (meta-analysis[Filter] OR review[Filter] OR systematicreview[Filter]) AND (fft[Filter]) AND (english[Filter])) | 2080 |
| **Search**: (water*[tw]) AND (southern africa*[tw])  **Filters**: Abstract, Free full text, Full text, Meta-Analysis, Review, Systematic Review, in the last 10 years, English  ("water*"[Text Word] AND "southern africa*"[Text Word]) AND ((y_10[Filter]) AND (ffrft[Filter]) AND (fha[Filter]) AND (meta-analysis[Filter] OR review[Filter] OR systematicreview[Filter]) AND (fft[Filter]) AND (english[Filter])) | 18 |
| **Search**: (sanitation*[tw]) AND (southern africa*[tw])  **Filters**: Abstract, Free full text, Full text, Meta-Analysis, Review, Systematic Review, in the last 10 years, English  ("sanitation*"[Text Word] AND "southern africa*"[Text Word]) AND ((y_10[Filter]) AND (ffrft[Filter]) AND (fha[Filter]) AND (meta-analysis[Filter] OR review[Filter] OR systematicreview[Filter]) AND (fft[Filter]) AND (english[Filter])) | 4 |
| **Search**: ((sanitation*[tw]) OR (shared sanitation*[tw])) AND (southern africa*)  **Filters**: Abstract, Free full text, Full text, Meta-Analysis, Review, Systematic Review, in the last 10 years, English  (("sanitation*"[Text Word] OR "shared sanitation*"[Text Word]) AND (("southern"[All Fields] OR "southerns"[All Fields]) AND "africa*"[All Fields])) AND ((y_10[Filter]) AND (ffrft[Filter]) AND (fha[Filter]) AND (meta-analysis[Filter] OR review[Filter] OR systematicreview[Filter]) AND (fft[Filter]) AND (english[Filter]))  **Translations**  southern: "southern"[All Fields] OR "southerns"[All Fields] | 4 |
| **Search**: (hygiene*[tw]) AND (southern africa*[tw])  **Filters**: Abstract, Free full text, Full text, Meta-Analysis, Review, Systematic Review, in the last 10 years, English  ("hygiene*"[Text Word] AND "southern africa*"[Text Word]) AND ((y_10[Filter]) AND (ffrft[Filter]) AND (fha[Filter]) AND (meta-analysis[Filter] OR review[Filter] OR systematicreview[Filter]) AND (fft[Filter]) AND (english[Filter])) | 1 |
| **Search**: (((hygiene*[tw]) OR (cleanliness*[tw])) AND (southern africa*[tw])) OR (africa*[tw])  **Filters**: Abstract, Free full text, Full text, Meta-Analysis, Review, Systematic Review, in the last 10 years, English  ((("hygiene*"[Text Word] OR "cleanliness*"[Text Word]) AND "southern africa*"[Text Word]) OR "africa*"[Text Word]) AND ((y_10[Filter]) AND (ffrft[Filter]) AND (fha[Filter]) AND (meta-analysis[Filter] OR review[Filter] OR systematicreview[Filter]) AND (fft[Filter]) AND (english[Filter])) | 9654 |
| **Search**: (((hygiene*[tw]) OR (cleanliness*[tw])) AND (southern africa*[tw])) OR (africa*[tw])  **Filters**: Abstract, Free full text, Full text, Meta-Analysis, Review, Systematic Review, English, from 2010/1/2 - 2021/12/31  ((("hygiene*"[Text Word] OR "cleanliness*"[Text Word]) AND "southern africa*"[Text Word]) OR "africa*"[Text Word]) AND ((ffrft[Filter]) AND (fha[Filter]) AND (meta-analysis[Filter] OR review[Filter] OR systematicreview[Filter]) AND (fft[Filter]) AND (2010/1/2:2021/12/31[pdat]) AND (english[Filter])) | 9931 |
