# Supplementary material for: Barriers and facilitators to Water, Sanitation and Hygiene (WaSH) practices in Southern Africa: A scoping review
Source: PLoS One. 2022 Aug 2;17(8):e0271726. doi: 10.1371/journal.pone.0271726 (PMC9345477; doi:10.1371/journal.pone.0271726)
Supplement: S2 File — (DOCX) [file pone.0271726.s003.docx]

**Quality of individual studies**

**For each study, scores ranged from 0 to 10, where 0 - 4 = ‘Low’, 5 – 7 = ‘Moderate’ and 8 – 10 = ‘High’.**

| **Article by author** | **Indicators** | **0** | **1** | **2** | **3** | **4** | **5** | **6** | **7** | **8** | **9** | **10** |
| --- | --- | --- | --- | --- | --- | --- | --- | --- | --- | --- | --- | --- |
|  | Clear definition of the study objective and aim |  |  |  |  |  |  |  | X |  |  |  |
|  | Study design appropriate for stated aims |  |  |  |  |  |  | X |  |  |  |  |
|  | Justified sample size |  |  |  |  |  | X |  |  |  |  |  |
| Tubatsi, G., Bonyongo, M.C. & Gondwe, M. (2015) – Botswana | Targeted population defined |  |  |  |  |  |  |  |  | X |  |  |
|  | Risk factor and outcome variables measured |  |  |  |  |  |  | X |  |  |  |  |
|  | Methods clearly described |  |  |  |  |  |  |  |  | X |  |  |
|  | Study results described |  |  |  |  |  |  |  | X |  |  |  |
|  | Discussions and conclusions justified |  |  |  |  |  |  |  | X |  |  |  |
|  | Study limitations discussed |  |  |  |  |  |  |  | X |  |  |  |
|  | Ethical approval for the study attained |  |  |  |  |  |  |  |  | X |  |  |
|  |  |  |  |  |  |  |  |  |  |  |  |  |
|  | Clear definition of the study objective and aim |  |  |  |  |  |  |  |  | **X** |  |  |
|  | Study design appropriate for stated aims |  |  |  |  |  |  |  | **X** |  |  |  |
|  | Justified sample size |  |  |  |  |  |  | **X** |  |  |  |  |
| McGill, B.M., Altchenko, Y., Hamilton, S.K., Kenabatho, P.K., Sylvester, S.R., & Villholth, K.G. (2019) – Botswana | Targeted population defined |  |  |  |  |  |  |  |  | **X** |  |  |
|  | Risk factor and outcome variables measured |  |  |  |  |  |  |  | **X** |  |  |  |
|  | Methods clearly described |  |  |  |  |  |  |  |  | **X** |  |  |
|  | Study results described |  |  |  |  |  |  |  | **X** |  |  |  |
|  | Discussions and conclusions justified |  |  |  |  |  |  |  | **X** |  |  |  |
|  | Study limitations discussed |  |  |  |  |  |  |  | **X** |  |  |  |
|  | Ethical approval for the study attained | **X** |  |  |  |  |  |  |  |  |  |  |
|  |  |  |  |  |  |  |  |  |  |  |  |  |
| **Article by author** | **Indicators** | **0** | **1** | **2** | **3** | **4** | **5** | **6** | **7** | **8** | **9** | **10** |
|  | Clear definition of the study objective and aim |  |  |  |  |  |  |  |  | X |  |  |
|  | Study design appropriate for stated aims |  |  |  |  |  |  |  |  | X |  |  |
|  | Justified sample size |  |  |  |  |  |  |  |  | X |  |  |
|  | Targeted population defined |  |  |  |  |  |  | X |  |  |  |  |
| Mlenga, D.H. (2016) - eSwadini | Risk factor and outcome variables measured |  |  |  |  |  |  | X |  |  |  |  |
|  | Methods clearly described |  |  |  |  |  |  |  |  | X |  |  |
|  | Study results described |  |  |  |  |  |  |  | X |  |  |  |
|  | Discussions and conclusions justified |  |  |  |  |  |  |  | X |  |  |  |
|  | Study limitations discussed | X |  |  |  |  |  |  |  |  |  |  |
|  | Ethical approval for the study attained | X |  |  |  |  |  |  |  |  |  |  |
|  |  |  |  |  |  |  |  |  |  |  |  |  |
| **Article by author** | **Indicators** | **0** | **1** | **2** | **3** | **4** | **5** | **6** | **7** | **8** | **9** | **10** |
|  | Clear definition of the study objective and aim |  |  |  |  |  |  |  | X |  |  |  |
|  | Study design appropriate for stated aims |  |  |  |  |  |  | X |  |  |  |  |
|  | Justified sample size |  |  |  |  |  | X |  |  |  |  |  |
|  | Targeted population defined |  |  |  |  |  |  | X |  |  |  |  |
| Gwimbi, P. (2011) - Lesotho | Risk factor and outcome variables measured |  |  |  |  |  | X |  |  |  |  |  |
|  | Methods clearly described |  |  |  |  |  |  | X |  |  |  |  |
|  | Study results described |  |  |  |  |  |  |  | X |  |  |  |
|  | Discussions and conclusions justified |  |  |  |  |  |  | X |  |  |  |  |
|  | Study limitations discussed | X |  |  |  |  |  |  |  |  |  |  |
|  | Ethical approval for the study attained | X |  |  |  |  |  |  |  |  |  |  |
|  |  |  |  |  |  |  |  |  |  |  |  |  |
| **Article by author** | **Indicators** | **0** | **1** | **2** | **3** | **4** | **5** | **6** | **7** | **8** | **9** | **10** |
|  | Clear definition of the study objective and aim |  |  |  |  |  |  | X |  |  |  |  |
|  | Study design appropriate for stated aims |  |  |  |  |  |  |  | X |  |  |  |
|  | Justified sample size |  |  |  |  |  |  | X |  |  |  |  |
|  | Targeted population defined |  |  |  |  |  |  |  |  | X |  |  |
| Gwimbi, P., George, M. & Ramphalile, M. (2019) - Lesotho | Risk factor and outcome variables measured |  |  |  |  |  |  | X |  |  |  |  |
|  | Methods clearly described |  |  |  |  |  |  |  | X |  |  |  |
|  | Study results described |  |  |  |  |  |  |  | X |  |  |  |
|  | Discussions and conclusions justified |  |  |  |  |  |  | X |  |  |  |  |
|  | Study limitations discussed |  | X |  |  |  |  |  |  |  |  |  |
|  | Ethical approval for the study attained |  |  |  |  |  |  |  | X |  |  |  |
|  |  |  |  |  |  |  |  |  |  |  |  |  |
| **Article by author** | **Indicators** | **0** | **1** | **2** | **3** | **4** | **5** | **6** | **7** | **8** | **9** | **10** |
|  | Clear definition of the study objective and aim |  |  |  |  |  | X |  |  |  |  |  |
|  | Study design appropriate for stated aims |  |  |  |  |  | X |  |  |  |  |  |
|  | Justified sample size |  |  |  |  |  |  |  | X |  |  |  |
| Chunga, R.M., Ensink, J.H.J., Jenkins, M.W. & Brown, J. (2016) - Malawi | Targeted population defined |  |  |  |  |  |  | X |  |  |  |  |
|  | Risk factor and outcome variables measured |  |  |  |  |  | X |  |  |  |  |  |
|  | Methods clearly described |  |  |  |  |  |  | X |  |  |  |  |
|  | Study results described |  |  |  |  |  |  |  | X |  |  |  |
|  | Discussions and conclusions justified |  |  |  |  |  |  | X |  |  |  |  |
|  | Study limitations discussed |  |  |  |  |  |  |  | X |  |  |  |
|  | Ethical approval for the study attained |  |  |  |  |  |  |  | X |  |  |  |
|  |  |  |  |  |  |  |  |  |  |  |  |  |
| **Article by author** | **Indicators** | **0** | **1** | **2** | **3** | **4** | **5** | **6** | **7** | **8** | **9** | **10** |
|  | Clear definition of the study objective and aim |  |  |  |  |  |  |  | X |  |  |  |
|  | Study design appropriate for stated aims |  |  |  |  |  |  | X |  |  |  |  |
|  | Justified sample size |  |  |  |  |  |  |  |  | X |  |  |
| Shiras, T., Cumming, O., Brown, J., Muneme, B., Nala, R. and Dreibelbis, R. (2018) – Mozambique | Targeted population defined |  |  |  |  |  |  |  | X |  |  |  |
|  | Risk factor and outcome variables measured |  |  |  |  |  |  | X |  |  |  |  |
|  | Methods clearly described |  |  |  |  |  |  | X |  |  |  |  |
|  | Study results described |  |  |  |  |  |  |  | X |  |  |  |
|  | Discussions and conclusions justified |  |  |  |  |  |  | X |  |  |  |  |
|  | Study limitations discussed |  |  |  |  |  |  |  | X |  |  |  |
|  | Ethical approval for the study attained |  |  |  |  |  |  |  |  | X |  |  |
|  |  |  |  |  |  |  |  |  |  |  |  |  |
| **Article by author** | **Indicators** | **0** | **1** | **2** | **3** | **4** | **5** | **6** | **7** | **8** | **9** | **10** |
|  | Clear definition of the study objective and aim |  |  |  |  |  |  | X |  |  |  |  |
|  | Study design appropriate for stated aims |  |  |  |  |  | X |  |  |  |  |  |
|  | Justified sample size |  |  |  |  |  |  | X |  |  |  |  |
|  | Targeted population defined |  |  |  |  |  | X |  |  |  |  |  |
| Hans-Joachim, M., Mosch, S. & Harter, M. (2018) – Mozambique | Risk factor and outcome variables measured. |  |  |  |  |  | X |  |  |  |  |  |
|  | Methods clearly described |  |  |  |  |  | X |  |  |  |  |  |
|  | Study results described |  |  |  |  |  |  | X |  |  |  |  |
|  | Discussions and conclusions justified |  |  |  |  |  |  | X |  |  |  |  |
|  | Study limitations discussed |  |  |  |  |  |  |  | X |  |  |  |
|  | Ethical approval for the study attained |  |  |  |  |  |  |  | X |  |  |  |
|  |  |  |  |  |  |  |  |  |  |  |  |  |
| **Article by author** | **Indicators** | **0** | **1** | **2** | **3** | **4** | **5** | **6** | **7** | **8** | **9** | **10** |
|  | Clear definition of the study objective and aim |  |  |  |  |  | X |  |  |  |  |  |
|  | Study design appropriate for stated aims |  |  |  |  |  | X |  |  |  |  |  |
|  | Justified sample size |  |  |  |  |  |  | X |  |  |  |  |
| Lewis, E.W., Nguza, S. & Selma, L. (2018) – Namibia | Targeted population defined |  |  |  |  |  |  |  | X |  |  |  |
|  | Risk factor and outcome variables measured |  |  |  |  |  |  | X |  |  |  |  |
|  | Methods clearly described |  |  |  |  |  | X |  |  |  |  |  |
|  | Study results described |  |  |  |  |  |  |  | X |  |  |  |
|  | Discussions and conclusions justified |  |  |  |  |  |  |  | X |  |  |  |
|  | Study limitations discussed | X |  |  |  |  |  |  |  |  |  |  |
|  | Ethical approval for the study attained | X |  |  |  |  |  |  |  |  |  |  |
|  |  |  |  |  |  |  |  |  |  |  |  |  |
| **Article by author** | **Indicators** | **0** | **1** | **2** | **3** | **4** | **5** | **6** | **7** | **8** | **9** | **10** |
|  | Clear definition of the study objective and aim |  |  |  |  |  |  |  |  | X |  |  |
|  | Study design appropriate for stated aims |  |  |  |  |  |  | X |  |  |  |  |
|  | Justified sample size |  |  |  |  |  |  | X |  |  |  |  |
|  | Targeted population defined |  |  |  |  |  |  |  |  | X |  |  |
| Abia, A.L.K., Schaefer, L., Ubomba-Jaswa, E., & Le Roux, W. (2017) South Africa | Risk factor and outcome variables measured. |  |  |  |  |  |  | X |  |  |  |  |
|  | Methods clearly described |  |  |  |  |  |  | X |  |  |  |  |
|  | Study results described |  |  |  |  |  |  |  | X |  |  |  |
|  | Discussions and conclusions justified |  |  |  |  |  |  |  | X |  |  |  |
|  | Study limitations discussed | X |  |  |  |  |  |  |  |  |  |  |
|  | Ethical approval for the study attained | X |  |  |  |  |  |  |  |  |  |  |
|  |  |  |  |  |  |  |  |  |  |  |  |  |
| **Article by author** | **Indicators** | **0** | **1** | **2** | **3** | **4** | **5** | **6** | **7** | **8** | **9** | **10** |
|  | Clear definition of the study objective and aim |  |  |  |  |  |  |  | X |  |  |  |
|  | Study design appropriate for stated aims |  |  |  |  |  |  | X |  |  |  |  |
|  | Justified sample size |  |  |  |  |  |  | X |  |  |  |  |
|  | Targeted population defined |  |  |  |  |  |  | X |  |  |  |  |
| Sibiya, J.E. & Gumbo, J.R. (2013) – South Africa | Risk factor and outcome variables measured |  |  |  |  |  | X |  |  |  |  |  |
|  | Methods clearly described |  |  |  |  |  |  |  | X |  |  |  |
|  | Study results described |  |  |  |  |  |  | X |  |  |  |  |
|  | Discussions and conclusions justified |  |  |  |  |  |  | X |  |  |  |  |
|  | Study limitations discussed | X |  |  |  |  |  |  |  |  |  |  |
|  | Ethical approval for the study attained |  |  |  |  |  | X |  |  |  |  |  |
|  |  |  |  |  |  |  |  |  |  |  |  |  |
| **Article by author** | **Indicators** | **0** | **1** | **2** | **3** | **4** | **5** | **6** | **7** | **8** | **9** | **10** |
|  | Clear definition of the study objective and aim |  |  |  |  |  |  | X |  |  |  |  |
|  | Study design appropriate for stated aims |  |  |  |  |  | X |  |  |  |  |  |
|  | Justified sample size |  |  |  |  |  | X |  |  |  |  |  |
|  | Targeted population defined |  |  |  |  |  | X |  |  |  |  |  |
| Nefale, A.D., Kamika, I., Obi, C.I. & Momba, M.N.B. (2017) – South Africa | Risk factor and outcome variables measured |  |  |  |  |  |  | X |  |  |  |  |
|  | Methods clearly described |  |  |  |  |  |  | X |  |  |  |  |
|  | Study results described |  |  |  |  |  |  |  | X |  |  |  |
|  | Discussions and conclusions justified |  |  |  |  |  |  | X |  |  |  |  |
|  | Study limitations discussed | X |  |  |  |  |  |  |  |  |  |  |
|  | Ethical approval for the study attained | X |  |  |  |  |  |  |  |  |  |  |
|  |  |  |  |  |  |  |  |  |  |  |  |  |
| **Article by author** | **Indicators** | **0** | **1** | **2** | **3** | **4** | **5** | **6** | **7** | **8** | **9** | **10** |
|  | Clear definition of the study objective and aim |  |  |  |  |  |  | X |  |  |  |  |
|  | Study design appropriate for stated aims |  |  |  |  |  |  |  | X |  |  |  |
|  | Justified sample size |  |  |  |  |  |  | X |  |  |  |  |
|  | Targeted population defined |  |  |  |  |  |  |  | X |  |  |  |
| Tidwell, J.B., Chipungu, J., Chilengi, R., Curtis, V. &  Aunger, R. (2019) – Zambia | Risk factor and outcome variables measured |  |  |  |  |  |  | X |  |  |  |  |
|  | Methods clearly described |  |  |  |  |  |  | X |  |  |  |  |
|  | Study results described |  |  |  |  |  |  |  | X |  |  |  |
|  | Discussions and conclusions justified |  |  |  |  |  |  | X |  |  |  |  |
|  | Study limitations discussed | X |  |  |  |  |  |  |  |  |  |  |
|  | Ethical approval for the study attained | X |  |  |  |  |  |  |  |  |  |  |
|  |  |  |  |  |  |  |  |  |  |  |  |  |
| **Article by author** | **Indicators** | **0** | **1** | **2** | **3** | **4** | **5** | **6** | **7** | **8** | **9** | **10** |
|  | Clear definition of the study objective and aim |  |  |  |  |  |  | X |  |  |  |  |
|  | Study design appropriate for stated aims |  |  |  |  |  |  | X |  |  |  |  |
|  | Justified sample size |  |  |  |  |  | X |  |  |  |  |  |
|  | Targeted population defined |  |  |  |  |  |  | X |  |  |  |  |
| Psutka, R., Peletz, R., Michelo, S., Kelly, P. & Clasen, T. (2020) – Zambia | Risk factor and outcome variables measured |  |  |  |  |  |  | X |  |  |  |  |
|  | Methods clearly described |  |  |  |  |  |  |  | X |  |  |  |
|  | Study results described |  |  |  |  |  |  |  | X |  |  |  |
|  | Discussions and conclusions justified |  |  |  |  |  |  |  | X |  |  |  |
|  | Study limitations discussed |  |  |  |  |  |  |  | X |  |  |  |
|  | Ethical approval for the study attained |  |  |  |  |  |  |  | X |  |  |  |
|  |  |  |  |  |  |  |  |  |  |  |  |  |
| **Article by author** | **Indicators** | **0** | **1** | **2** | **3** | **4** | **5** | **6** | **7** | **8** | **9** | **10** |
|  | Clear definition of the study objective and aim |  |  |  |  |  |  | X |  |  |  |  |
|  | Study design appropriate for stated aims |  |  |  |  |  |  | X |  |  |  |  |
|  | Justified sample size |  |  |  |  |  |  |  | X |  |  |  |
|  | Targeted population defined |  |  |  |  |  |  |  | X |  |  |  |
| Thys, S., Mwape, K.E., Lefèvre, P., Dorny, P., Marcotty, T.,  Phiri, A.M., Phiri, I.K. & Gabriël, S. (2015) – Zambia | Risk factor and outcome variables measured |  |  |  |  |  |  | X |  |  |  |  |
|  | Methods clearly described |  |  |  |  |  |  |  | X |  |  |  |
|  | Study results described |  |  |  |  |  |  | X |  |  |  |  |
|  | Discussions and conclusions justified |  |  |  |  |  |  | X |  |  |  |  |
|  | Study limitations discussed | X |  |  |  |  |  |  |  |  |  |  |
|  | Ethical approval for the study attained |  |  |  |  |  |  |  | X |  |  |  |
|  |  |  |  |  |  |  |  |  |  |  |  |  |
| **Article by author** | **Indicators** | **0** | **1** | **2** | **3** | **4** | **5** | **6** | **7** | **8** | **9** | **10** |
|  | Clear definition of the study objective and aim |  |  |  |  |  | X |  |  |  |  |  |
|  | Study design appropriate for stated aims |  |  |  |  |  |  |  | X |  |  |  |
|  | Justified sample size |  |  |  |  |  |  | X |  |  |  |  |
|  | Targeted population defined |  |  |  |  |  |  |  | X |  |  |  |
| Tidwell, J.B., Chipungu, J., Bosomprah, S., Aunger, R., Curtis, V. & Chilengi, R. (2019) – Zambia | Risk factor and outcome variables measured |  |  |  |  |  |  |  | X |  |  |  |
|  | Methods clearly described |  |  |  |  |  |  | X |  |  |  |  |
|  | Study results described |  |  |  |  |  |  |  | X |  |  |  |
|  | Discussions and conclusions justified |  |  |  |  |  |  | X |  |  |  |  |
|  | Study limitations discussed | X |  |  |  |  |  |  |  |  |  |  |
|  | Ethical approval for the study attained |  |  |  |  |  |  |  | X |  |  |  |
|  |  |  |  |  |  |  |  |  |  |  |  |  |
| **Article by author** | **Indicators** | **0** | **1** | **2** | **3** | **4** | **5** | **6** | **7** | **8** | **9** | **10** |
|  | Clear definition of the study objective and aim |  |  |  |  |  |  | X |  |  |  |  |
|  | Study design appropriate for stated aims |  |  |  |  |  |  | X |  |  |  |  |
|  | Justified sample size |  |  |  |  |  |  | X |  |  |  |  |
|  | Targeted population defined |  |  |  |  |  |  | X |  |  |  |  |
| Yeboah-Antwi, K., MacLeod, W.B., Biemba, G., Sijenyi, P., Hohne, A., Verstraete, L.,  McCallum, C.M. & Hamer, D.H. (2019) – Zambia | Risk factor and outcome variables measured |  |  |  |  |  | X |  |  |  |  |  |
|  | Methods clearly described |  |  |  |  |  |  |  | X |  |  |  |
|  | Study results described |  |  |  |  |  |  | X |  |  |  |  |
|  | Discussions and conclusions justified |  |  |  |  |  |  | X |  |  |  |  |
|  | Study limitations discussed |  |  |  |  |  |  |  | X |  |  |  |
|  | Ethical approval for the study attained |  |  |  |  |  |  |  | X |  |  |  |
|  |  |  |  |  |  |  |  |  |  |  |  |  |
| **Article by author** | **Indicators** | **0** | **1** | **2** | **3** | **4** | **5** | **6** | **7** | **8** | **9** | **10** |
|  | Clear definition of the study objective and aim |  |  |  |  |  |  |  | X |  |  |  |
|  | Study design appropriate for stated aims |  |  |  |  |  |  |  | X |  |  |  |
|  | Justified sample size |  |  |  |  |  |  | X |  |  |  |  |
|  | Targeted population defined |  |  |  |  |  |  | X |  |  |  |  |
| Ncube, F., Kanda, A., Chahwanda, M., Margaret Macherera, M. & Ngwenya, B. (2020) – Zimbabwe | Risk factor and outcome variables measured |  |  |  |  |  |  | X |  |  |  |  |
|  | Methods clearly described |  |  |  |  |  |  |  | X |  |  |  |
|  | Study results described |  |  |  |  |  |  |  |  | X |  |  |
|  | Discussions and conclusions justified |  |  |  |  |  |  |  | X |  |  |  |
|  | Study limitations discussed |  |  |  |  |  |  |  | X |  |  |  |
|  | Ethical approval for the study attained | X |  |  |  |  |  |  |  |  |  |  |
