# Supplementary material for: Barriers and facilitators to Water, Sanitation and Hygiene (WaSH) practices in Southern Africa: A scoping review
Source: PLoS One. 2022 Aug 2;17(8):e0271726. doi: 10.1371/journal.pone.0271726 (PMC9345477; doi:10.1371/journal.pone.0271726)
Supplement: S1 Protocol — (DOCX) [file pone.0271726.s004.docx]

**Protocol**

| **Title** | Facilitators and barriers to water, sanitation, and hygiene (WaSH) practices in Southern Arica: a systematic review | |
| --- | --- | --- |
| **Purpose** | To examine facilitators and barriers to effective WaSH practices, and to identify research gaps on facilitators and barriers to effective WaSH practices in Southern Africa and describe direction for future research. | |
| **Research question** | What are the facilitators and barriers to effective water, sanitation, and hygiene (WaSH) practices in Southern Africa? | |
| **Specific objectives** | - To synthesize the existing information on WaSH practices in Southern Africa. - To examine facilitators to effective WaSH practices in Southern Africa. - To identify the existing research gaps on facilitators and barriers to effective WaSH practices in Southern African countries. | |
| **Keywords** | - Facilitators and barriers - Water, sanitation, and hygiene - WASH practices - Southern Africa | |
| **Synonyms** | **Facilitators** | Drivers, motivators, enablers |
|  | **Barriers** | Challenges, hindrances, obstacles |
| **Sources/digital libraries** | - PubMed - Medline - EbscoHost - Google Scholar | |
| **Search strings** | We will conduct a systematic electronic search of peer reviewed journal articles from various databases including Google Scholar, PubMed, EbscoHost and Medline using the following keywords: “facilitators; barriers; water; sanitation; hygiene; WaSH practices and Southern Africa.” Using the keywords, we developed “index terms” from combining keywords and their synonyms and used the Boolean operators “AND”, “OR” and truncations to create search strings: “Water AND sanitation AND hygiene AND Facilitators (AND motivators) AND barriers (OR hindrances) AND WASH practices AND Southern Africa”. | |
| **Inclusion criteria** | - Peer reviewed journal articles - qualitative, quantitative, and mixed methods studies on facilitators and barriers to WaSH practices in Southern Africa. - Publications from 2010 – June 2021. - Studies describing WaSH practices in Southern African countries (Angola, Botswana, Lesotho, Mozambique, Namibia, South Africa, Swaziland, Zambia, Zimbabwe). - Publication language: English | |
| **Exclusion criteria** | - Publications before 2010. - Studies describing WaSH practices in other continents outside Southern Africa. - Publications in other language other than English. - Old reviews, e.g., systematic, scoping and meta-analysis (published before 2010). | |
