# Supplementary material for: Barriers and facilitators to Water, Sanitation and Hygiene (WaSH) practices in Southern Africa: A scoping review
Source: PLoS One. 2022 Aug 2;17(8):e0271726. doi: 10.1371/journal.pone.0271726 (PMC9345477; doi:10.1371/journal.pone.0271726)
Supplement: Attachment — Submitted filename: Scoping review_PONE-D-21-26335.docx [file pone.0271726.s005.docx]

**Manuscript Number**: PONE-D-21-26335

**Full Title:** Barriers and facilitators to Water, Sanitation and Hygiene (WaSH) practices in Southern Africa: a scoping review

**Short Title:** Barriers and facilitators to Water, Sanitation and Hygiene practices in Southern Africa:a scoping reviews

**General Comment**

1. **“** Rural areas are the most affected by barriers to improved WaSH facilities compared to urban settings.

Is that population growth is a barrier for rural set up or urban in southern Africa? One of the team is population growth,

**Specific Comment**

**Abstract:**

2. “ The general themes that emerged included geographical inequalities, climate change, investment on WaSH resources, low levels of knowledge on water borne diseases and ineffective local community engagement”

Is this general for barriers and facilitator for WASH, please it need to specify and indicate clearly for the reader?

**Introduction:**

3. The second paragraph in References citation indicated that “Inadequate water, access to improved sanitation, and hygiene (WaSH) are global health challenges affecting about one-third of the world’s population [8, 10].” since, the reference system style of the journal is Vancouver (number system) , better start with 1, then 2 , 3.4…..in an ascending manner. rather than start with 8? Like [8, 10] indicated the manuscript.
4. Paragraph 3 line four, “ However, people still lack adequate information on WaSH leading to poor sanitation and hygiene practices.” It needs citation or evidence to this sentence?

**Method**

5. Inclusion and Exclusion section “ We excluded reviews, i.e. systematic, scoping and meta-analysis.” It is not clear that, was there any systematic review and metanalysis in similar topic in the study setting? if that is the case, what is the importance of this review? How many did you get the three systematic, scoping, and metanalysis?

**Result**

6. In the eligibility criteria; “ Five (5) additional records were identified from the reference lists of eligible articles and were included for full text review” This is not clear for your inclusion, is that a grey literature ? or you already acknowledge as a limitation as included only published articles?

Discussion

7. In the first summary paragraph “The following barrier themes emerged from the analysis: (1) geographical inequalities, (2) climate change, (3) low investment on WaSH infrastructure, (4) low knowledge levels on waterborne diseases, (5) ineffective local community engagement.

In the data abstraction sheet both barriers and facilitators are put clearly, while in this paragraph Please also show the facilitators in this summary finding paragraph?
