# Supplementary material for: Barriers and facilitators to Water, Sanitation and Hygiene (WaSH) practices in Southern Africa: A scoping review
Source: PLoS One. 2022 Aug 2;17(8):e0271726. doi: 10.1371/journal.pone.0271726 (PMC9345477; doi:10.1371/journal.pone.0271726)
Supplement: Attachment — Submitted filename: Response to Reviewers.docx [file pone.0271726.s006.docx]

**The rebuttal letter**

**Manuscript Number**: PONE-D-21-26335

**Full Title:** Barriers and facilitators to Water, Sanitation and Hygiene (WaSH) practices in Southern Africa: a scoping review

**Short Title:** Barriers and facilitators to Water, Sanitation and Hygiene practices in Southern Africa: a scoping review

| **Reviewer’s comments** | **The authors’ response** | **Page in the manuscript** |
| --- | --- | --- |
| **General Comment**   1. **“Rural** areas are the most affected by barriers to improved WaSH facilities compared to urban settings.   Is that population growth is a barrier for rural set up or urban in southern Africa? One of the team is population growth, | Population growth is mostly a barrier for service delivery in urban areas in South Africa, however, with regards to WaSH facilities, most South African rural settings lack WaSH infrastructure resulting in these areas as the most negatively affected barriers to improved WaSH facilities.  Rural areas, due to lack of WaSH infrastructure are the most affected by barriers to improved WaSH facilities compared to urban settings. | Page 1 |
| **Specific Comment**  **Abstract:**   2. “The general themes that emerged included geographical inequalities, climate change, investment on WaSH resources, low levels of knowledge on water borne diseases and ineffective local community engagement”   Is this general for barriers and facilitator for WASH, please it need to specify and indicate clearly for the reader? | The general themes that emerged for both barriers and facilitators included geographical inequalities, climate change, investment on WaSH resources, low levels of knowledge on water borne diseases and ineffective local community engagement. | Page 1 |
| **Introduction:**   3. The second paragraph in References citation indicated that “Inadequate water, access to improved sanitation, and hygiene (WaSH) are global health challenges affecting about one-third of the world’s population [8, 10].” since, the reference system style of the journal is Vancouver (number system), better start with 1, then 2, 3.4…..in an ascending manner. rather than start with 8? Like [8, 10] indicated the manuscript. | The references have been re-ordered to follow an ascending order in the manuscript and the reference list. | Page 1 – 20 |
| 4. Paragraph 3 line four, “However, people still lack adequate information on WaSH leading to poor sanitation and hygiene practices.” It needs citation or evidence to this sentence? | However, people still lack adequate information on WaSH leading to poor sanitation and hygiene practices [8]. | Page 2 |
| **Method**   5. Inclusion and Exclusion section: “We excluded reviews, i.e. systematic, scoping and meta-analysis.” It is not clear that, was there any systematic review and metanalysis in similar topic in the study setting? if that is the case, what is the importance of this review? How many did you get the three systematic, scoping, and metanalysis? | There were no reviews, i.e. systematic, scoping and metanalysis with similar topic in the study setting.  We excluded reviews, i.e. systematic, scoping and meta-analysis that were published before 2010. | Page 4 |
| **Result**   6. In the eligibility criteria; “Five (5) additional records were identified from the reference lists of eligible articles and were included for full text review”. This is not clear for your inclusion, is that a grey literature? or you already acknowledge as a limitation as included only published articles?   **Discussion**   7. In the first summary paragraph “The following barrier themes emerged from the analysis: (1) geographical inequalities, (2) climate change, (3) low investment on WaSH infrastructure, (4) low knowledge levels on waterborne diseases, (5) ineffective local community engagement.   In the data abstraction sheet both barriers and facilitators are put clearly, while in this paragraph Please also show the facilitators in this summary finding paragraph? | Five (5) additional records were identified from the reference lists of eligible articles and were included as grey literature for full text review.  Facilitators for WaSH practices that emerged from the analysis included: (a) effective local community’s engagement in WaSH interventions, (b) increased investment on WaSH infrastructure, (c) local community’s engagement in WaSH interventions, (d) increased latrine ownership and (e) development of social capital within small community units. | Page 5  Page 11 |
| **Comments from the Journal (Journal Requirements)** |  |  |
| Please upload a new copy of Figure 1 as the detail is not clear. | A clearer copy of Figure 1 has replaced the old unclear figure. | Page 5 |
| Please review your reference list to ensure that it is complete and correct. | The reference list has been reviewed and re-ordered both in the manuscript and the reference list to match the reference style of the journal - the Vancouver (number system). References are now ordered in an ascending order. | Page 1 -20 |
